# Streptococcal Toxic Shock Syndrome: A Case Report Highlighting the Importance of Early Recognition and Intervention

**DOI:** 10.7759/cureus.89562

**Published:** 2025-08-07

**Authors:** Molly E Boyko

**Affiliations:** 1 Internal Medicine, St Luke's Hospital, Easton, USA

**Keywords:** case report, cellulitis, severity of cellulitis, soft-tissue infection, streptococcal bacteremia, streptococcal toxic shock syndrome, stss treatment

## Abstract

Streptococcal toxic shock syndrome (STSS) is a rare but life-threatening illness characterized by rapid progression to multi-organ failure. This is a case of a middle-aged male patient who initially presented with localized chest wall pain, erythema, vomiting, and diarrhea. These nonspecific symptoms rapidly progressed to systemic shock and multi-organ dysfunction, including acute kidney injury, pleural effusions, demand ischemia of the heart, and the development of a characteristic diffuse, sunburn-like rash. The likely inciting factor was a soft-tissue infection of the chest wall. The patient was treated with broad-spectrum intravenous antibiotics and supportive care in the intensive care unit and step-down unit. Following stabilization after the acute phase, the patient was readmitted due to complications and faced a prolonged recovery. Early symptoms are often vague, and many cases are initially misdiagnosed, highlighting the importance of early diagnostic consideration. This case demonstrates the impact of prompt recognition and timely intervention in STSS. Additionally, it emphasizes the importance of ongoing follow-up due to the risk of long-term sequelae. Clinicians should maintain a high index of suspicion when evaluating patients with rapidly progressing soft-tissue infections and systemic signs.

## Introduction

Streptococcal toxic shock syndrome (STSS) is a rare, life-threatening condition often triggered by invasive group A *Streptococcus* (GAS) infections. The term GAS is often used synonymously with *Streptococcus pyogenes*, the most common type of GAS, but the group also includes *Streptococcus dysgalactiae* and some strains of *Streptococcus anginosus* [1]. It is characterized by rapid progression to multi-organ failure, shock, and systemic toxicity, often in association with soft-tissue infections such as cellulitis. In at least half of the cases, the portal of entry for streptococci cannot be definitively identified, and in many other cases, it can only be presumed [2]. Various experimental studies suggest that streptococcal superantigens play a crucial role as virulence factors in severe streptococcal infections. SpeA and SpeC are the primary streptococcal superantigens, and these toxins are considered the likely cause of most cases of STSS [3]. Invasive streptococcal disease occurs at an incidence of 3.5 cases per 100,000 individuals, with a high mortality rate [4].

STSS is typically described in three phases. The first phase, which occurs 24 to 48 hours before the onset of severe hypotension, is marked by a severe influenza-like illness. This phase is characterized by high fever, myalgia, headaches, and chills. Approximately 20% of patients present with an influenza-like illness [2]. Additionally, nonspecific gastrointestinal symptoms, such as nausea, vomiting, and diarrhea, may also be present during this early stage [5]. The second phase of STSS is marked by systemic symptoms, including high fever, tachypnea, and tachycardia. The third phase of clinical presentation is characterized by sudden and severe circulatory shock, often accompanied by multiple organ failure [5]. Other characteristic clinical features include fever ≥ 102°F, renal impairment, and desquamating rash [1]. The standard of care is to promptly initiate empiric antibiotic therapy in suspected STSS due to its clinical overlap with other sepsis syndromes. Initial broad-spectrum coverage is essential and should target GAS, *Staphylococcus aureus* (including methicillin-resistant *S. aureus* (MRSA)), and gram-negative bacilli. A recommended empiric regimen includes clindamycin plus vancomycin, in combination with either a carbapenem or a beta-lactam/beta-lactamase inhibitor. Therapy should be refined once culture results are available [6]. Once STSS is confirmed, targeted antibiotic therapy should include high-dose penicillin G or ceftriaxone plus clindamycin. This combination optimizes bacterial killing and suppresses toxin production, which is critical in managing severe GAS infections [6]. Intravenous immunoglobulin (IVIG), hyperbaric oxygen therapy, and anti-tumor necrosis factor (TNF) antibodies are adjunctive therapies for treatment [6]. While prompt recognition and intervention can significantly improve outcomes, the subtle initial presentation can sometimes lead to misdiagnosis or premature discharge from care. The patient in this case was initially misdiagnosed with gastroenteritis, which is a common occurrence given the vague and nonspecific presenting symptoms that often precede the identification of bacteremia. Other frequent early misdiagnoses include viral illness, urinary tract infection, cellulitis, and sepsis of unknown origin. This case highlights the critical importance of recognizing early signs of STSS, particularly in patients with skin findings, and the need for thorough, continued treatment before considering discharge.

## Case presentation

A 49-year-old man with a history of pulmonary embolism and deep venous thrombosis on apixaban, and narcolepsy, presented to an outside facility with a three-day history of nausea, vomiting, diarrhea, and right neck and chest wall pain. He was diagnosed with viral gastroenteritis and discharged with a prescription for ondansetron. The following day, he returned to the facility with an acute onset of chills, fever, generalized body aches, and persistent nausea, vomiting, and diarrhea. On physical examination, the patient appeared acutely ill, in distress, tachycardic, tachypneic, and hypotensive. He also had an erythematous rash on his right neck and chest wall.

Given evidence of sepsis, acute renal failure, and hypotension requiring pressor support, the patient was admitted to the intensive care unit (ICU). Blood cultures were obtained and grew *S. pyogenes*, a gram-positive cocci. He was diagnosed with STSS and was initially treated with vancomycin and piperacillin-tazobactam, which was later narrowed to ceftriaxone and clindamycin. The patient also received IVIG. His symptoms improved, renal function returned to baseline, and repeat blood cultures were negative. The hospital stay was three days, and he was discharged with an 11-day course of amoxicillin/clavulanic acid and advised to follow up with his primary care physician.

**Figure 1:**
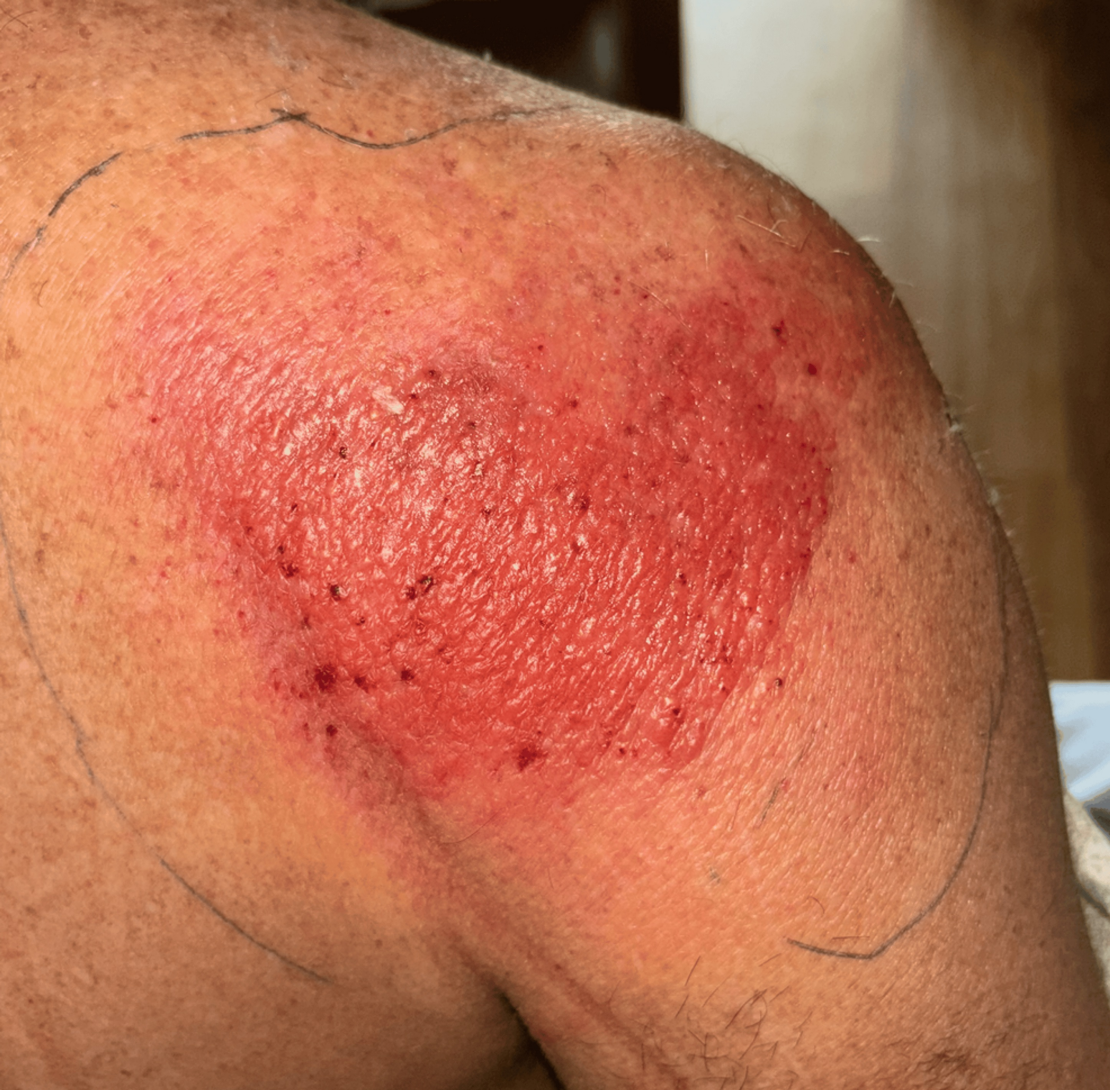
A 10 cm x 10 cm poorly demarcated, diffusely erythematous rash on the right posterior shoulder

Two days after being discharged from the previous facility, the patient’s neck, right arm, and shoulder pain worsened, and he developed shortness of breath and chest pain, prompting his return to a different emergency department. On day one of his second hospitalization, physical examination revealed tachypnea, though other vital signs were within normal limits. A mildly erythematous, exfoliative rash with areas of scaling was noted on the right shoulder, which was tender to palpation but dry, intact, and without urticaria, bullae, vesicles, significant induration, or fluctuance (Figure 1).

The patient underwent further evaluation, including an ultrasound of his extremity, which was negative for deep venous thrombosis and other pathologies. A magnetic resonance imaging (MRI) of the neck revealed no acute findings, and an electrocardiogram (EKG) was normal. Chest X-ray showed progressive bilateral perihilar and basilar airspace opacities along with trace pleural effusions. A computed tomography (CT) scan of the abdomen and pelvis revealed new fluid third-spacing, mild anasarca, and small-volume free intraperitoneal fluid. A pulmonary embolism was ruled out. Notably, the patient had a significantly elevated white blood cell count (WBC) with neutrophil predominance and elevated C-reactive protein (CRP) (Table 1). He was treated with morphine, ondansetron, acetaminophen, ketorolac, furosemide, clindamycin, and ceftriaxone. Blood cultures were collected and remained negative for growth over a five-day period.

**Table 1:** Key laboratory values on admission, hospital day 3, and day of discharge (day 6) WBC: white blood cell count; CRP: C-reactive protein; AST: aspartate aminotransferase; ALT: alanine aminotransferase

Parameter	Initial values	Repeated values	Values on discharge	Units
WBC	20.33	12.84	6.32	10^3^/µL
Segmented	88	72	66	%
CRP	7.80	6.60	0.89	mg/dL
Creatinine	2.27	2.09	1.1	mg/dL
AST	94	59	47	U/L
ALT	80	71	55	U/L
Troponin	22	17	Not collected	ng/L

On day two of his second hospitalization, the patient's condition and labs remained mostly stable. Notably, his WBC decreased and troponin increased. Cardiology was consulted due to the elevated troponin and suspected demand ischemia in the context of the acute infection. A transthoracic echocardiogram was recommended to assess for vegetations and cardiac structure and function, which returned normal.

On day three of his second hospitalization, the patient's pain worsened, particularly with movement, and his shortness of breath became more pronounced, prompting a consult with pulmonology. A repeat ultrasound revealed small-caliber effusion pockets with lung parenchyma very close to the chest wall. Given the bilateral nature of the effusions and their simple architecture, without loculations or other signs of empyema, pulmonology was not suspicious for a new infection but rather attributed it to previous toxic shock syndrome or septic fluid resuscitation. Additionally, perifissural fluid suggested volume overload. The patient's WBC continued to decrease.

On day four, the patient's clinical signs and symptoms remained consistent with the previous day. However, on day five, he began experiencing right upper quadrant (RUQ) and right flank pain, accompanied by tenderness upon deep palpation of the RUQ. A CT scan of the abdomen and pelvis revealed splenomegaly. A hepatobiliary iminodiacetic acid (HIDA) scan showed normal gallbladder filling and ejection. Additionally, a blanching erythematous rash started to spread across his face and trunk, and he reported widespread myalgia. Given these developments, the differential diagnosis was expanded to include dengue virus, Epstein-Barr virus, familial Mediterranean fever, and tick-borne diseases. Serology was ordered for each condition, all of which returned negative results. The primary team concluded that the sequelae the patient experienced were attributable to the STSS and streptococcal bacteremia.

On day six, the patient showed notable clinical improvement, with all abnormal lab results returning to baseline. After a thorough review of lab work and imaging, which revealed no new diagnoses and demonstrated ongoing improvement, he was deemed stable and ready for discharge. Clindamycin was discontinued, and the patient was discharged with cephalexin to complete a 14-day total course and was not readmitted for this condition.

## Discussion

Limitations in the approach to this case include the fact that the patient initially presented and received ICU-level care at an outside facility. As a result, there was no direct involvement in the early management or ability to observe the acute phase of illness in real time. Clinical history and details of the initial presentation were obtained through thorough chart review, with the patient's consent, as well as through discussions with the patient. While every effort was made to ensure accuracy, the lack of direct observation may limit the completeness of some clinical details.

A key strength of this case is that it opens discussion on the sequelae of STSS. Direct involvement in the patient's care during this phase allowed for observation of how ongoing complications prolonged the patient's hospitalization and necessitated further diagnostic workup, including additional labs, imaging, and consultations. This experience highlights the critical importance of early recognition and appropriately tailored treatment, not only to manage the acute illness but also to reduce the risk and burden of long-term complications.

Early recognition of STSS is essential to improving patient outcomes, as the condition can progress rapidly and lead to multi-organ failure. The patient in this case displayed some, but not all, of the typical initial symptoms of STSS, including chills, fever, and generalized body aches, as well as persistent nausea, vomiting, and diarrhea. However, his gastrointestinal symptoms, combined with chest wall pain, should have raised concerns about a potential skin infection, which could have indicated the onset of the early phase of STSS. A thorough examination of the skin and soft tissues is crucial during the initial clinical evaluation of patients, as well as throughout follow-up care [5]. Skin rashes can be challenging to characterize, as demonstrated in this case, where differential diagnoses such as tick-borne illness were considered. Over time, the rash extended to the trunk and face, becoming more maculopapular and erythematous. It did not correspond with the typical presentation or distribution of a tick-borne illness, leading to the conclusion that it was a sequela of STSS.

In 2010, the CDC established a specific set of guidelines for diagnosing STSS, outlined as follows: hypotension and multi-organ involvement characterized by at least two of the following: renal impairment, coagulopathy, liver dysfunction, total bilirubin greater than twice the upper limit of normal, acute respiratory distress syndrome, a generalized erythematous macular rash, and soft-tissue necrosis [7]. These criteria are important to consider when a patient presents with nonspecific flu-like symptoms. Healthcare providers should assess whether the patient meets these criteria to ensure that treatment is initiated promptly, given the high mortality rate associated with delayed intervention.

Equally important as early disease recognition is the proper treatment regimen and duration. Effective management of septic physiology, including fluid resuscitation, vasopressor support, surgical debridement, and the administration of appropriate antibiotics, forms the cornerstone of treatment [7]. Once the patient was diagnosed with streptococcal bacteremia and STSS, all appropriate therapies were initiated, except for surgical debridement, as there was no evidence of necrotizing fasciitis. Ideally, the severity of this condition should be recognized during initial presentation, as early suspicion and intervention are crucial for survival in infections with a high mortality rate [8].

Although GAS is susceptible to penicillin, studies have shown significantly better outcomes in cases of STSS when clindamycin and IVIG are added to beta-lactam antibiotic treatment [5]. There are no clinical studies that define the optimal duration of antibiotic therapy for STSS [6]. The length of treatment should be customized based on individual patient factors, such as the source of infection and response to therapy. For patients with bacteremia, antibiotic treatment should generally last at least 14 days [6]. Adjunctive therapies for treating invasive GAS infections include IVIG, hyperbaric oxygen, and anti-TNF antibodies. In this case, the patient received IVIG, a treatment supported by a 2018 meta-analysis of five studies on patients with STSS treated with clindamycin (one randomized and four nonrandomized). The analysis found that IVIG use was linked to a reduction in 30-day mortality [6].

## Conclusions

STSS is a rapidly progressing and life-threatening condition that can affect previously healthy individuals. Early recognition, a high index of suspicion, and aggressive intervention are crucial to improving outcomes. The severity of STSS, driven by superantigen activity from GAS, leads to rapid organ failure and potential tissue necrosis, making it essential to provide prompt, multi-disciplinary care. Effective treatment involves rapid administration of broad-spectrum antibiotics, including beta-lactams and clindamycin, as well as surgical debridement in cases of necrotizing fasciitis. Further therapeutic strategies, such as immunoglobulins and hyperbaric oxygen, may hold promise in reducing the high mortality associated with this syndrome. Many cases are misdiagnosed in the early stages due to vague presentations, making early diagnostic consideration essential. This case illustrates the critical role of early recognition and timely intervention in STSS, given its high risk of mortality.
